# Serological survey of SARS-CoV-2 in companion animals in China

**DOI:** 10.3389/fvets.2022.986619

**Published:** 2022-11-30

**Authors:** Aiping Wang, Xifang Zhu, Yumei Chen, Yaning Sun, Hongliang Liu, Peiyang Ding, Jingming Zhou, Yankai Liu, Chao Liang, Jiajia Yin, Gaiping Zhang

**Affiliations:** ^1^School of Life Sciences, Zhengzhou University, Zhengzhou, China; ^2^Henan Zhongze Biological Engineering Co., Ltd., Zhengzhou, China

**Keywords:** SARS-CoV-2, seroprevalence, companion animals, ELISA, colloidal gold test strips

## Abstract

Severe Acute Respiratory Syndrome Coronavirus 2 (SARS-CoV-2) can be transmitted from human to companion animals. The national wide serological surveillance against SARS-CoV-2 was conducted among pet animals, mainly in cats and dogs, 1 year after the first outbreak of COVID-19 in China. All sera were tested for SARS-CoV-2 IgG antibodies using an indirect enzyme linked immunosorbent assay (ELISA) based on the receptor binding domain (RBD) of spike protein. This late survey takes advantage of the short duration of the serological response in these animals to track recent episode of transmission. A total of 20,592 blood samples were obtained from 25 provinces across 7 geographical regions. The overall seroprevalence of SARS-CoV-2 infections in cats was 0.015% (2/13397; 95% confidence intervals (CI): 0.0, 0.1). The virus infections in cats were only detected in Central (Hubei, 0.375%) and Eastern China (Zhejiang, 0.087%) with a seroprevalence estimated at 0.090 and 0.020%, respectively. In dogs, the seroprevalence of SARS-CoV-2 infections was 0.014% (1/7159; 95% CI: 0.0, 0.1) in the entire nation, seropositive samples were limited to Beijing (0.070%) of Northern China with a prevalence of 0.054%. No seropositive cases were discovered in other geographic regions, nor in other companion animals analyzed in this study. These data reveal the circulation of SARS-CoV-2 in companion animals, although transmission of the virus to domestic cats and dogs is low in China, continuous monitoring is helpful for the better understand of the virus transmission status and the effect on animals.

## Introduction

The COVID-19 (Coronavirus Disease 2019) pandemic seriously threaten the global public health. The typical symptoms of this disease are fever, cough, difficulty breathing, severe pneumonia, and even death (1). According to the coronavirus dashboard of World Health Organization (WHO), the confirmed cases and deaths reached 542,188,789 and 6,329,275, respectively, while the confirmed cases and death in China were 126,384 and 5,696, respectively until June 29, 2022 (2). The main cause of severe symptomatic cases and deaths is attributed to cytokines storm and the subsequent leading of acute respiratory distress syndrome (ARDS) (3).

Angiotensin-converting enzyme II (ACE2) is the receptor of spike (S) glycoprotein during SARS-CoV-2 infection (4, 5). Protein sequences and structural modeling analysis revealed that ACE2 is widely distributed in animal species, representing a potential risk of cross-species transmission for this virus (6, 7). SARS-CoV-2 infection has been reported in companion animals including cats, dogs, rabbits, ferrets and minks (8, 9). Animal experimental infections indicate that cats can infect SARS-CoV-2. Virus inoculated cats seroconverted against S, nucleocapsid (N) and RBD antigens within 5–7 days, and the specific antibody titer reached the peak at day 14. Moreover, the virus can be efficiently transmitting between cats, animals exposed to inoculated cats can produce specific antibodies against S and N proteins within 2 weeks (10, 11). However, the duration of the serological response in companion animals is still poorly documented, antibodies titer against RBD protein reached the peak on day 10 in natural infected cats and decreased to the detection limitation within 110 days (12). The susceptibility to SARS-CoV-2 are relatively lower in dogs. Experimental infections showed IgG antibodies against S and RBD proteins were detectable at day 14, and reached plateau or start to decrease by day 42 (11). In Europe, the seroprevalence of SARS-CoV-2 infection in companion animals was found to range from 0.4 to 3.5% in cats and 0.2 to 3.3% in dogs (13–17). In minks infected farms, SARS-CoV-2 transmitted to cats and dogs were analyzed by PCR and whole genome sequencing, revealing a mink-to-cat transmitting rate of 12%, but no mink-to-dog spread happened (18). Experimental infections confirmed the ferret-to-ferret spread of SARS-CoV-2. The virus RNA could be detected from nasal washes and fecal in both directly contact group and indirectly contact group within 4 days, while the viral RNA in saliva and urine was detected only in the directly contact group (19). Recent studies have documented infection cases in pet ferrets and ferrets back to human propagation events (20, 21).

Several serological test methods have been used to detect IgG and/or IgM antibodies against SARS-CoV-2 infection in humans (22–25). Recently, an indirect ELISA and a multi-species ELISA have been developed to detect antibodies against SARS-CoV-2 in several animal species by using RBD as antigen (17, 26–29).

The objective of this study was to investigate the SARS-CoV-2 seroprevalence in companion animals 1 year after the first outbreak in China. This “late” serological survey can provide useful information on the current rate of transmission between human and companion animals.

## Materials and methods

### Sample collections

**Figure 1:**
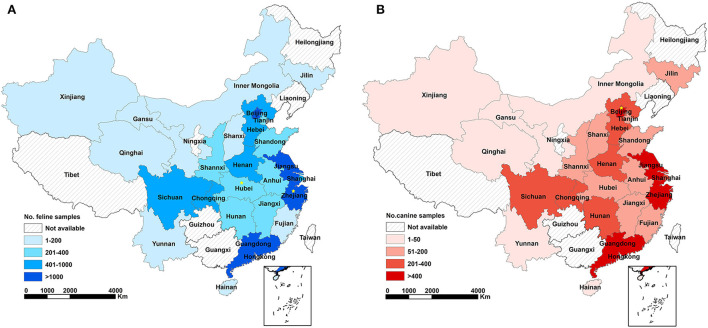
Geographical coverage of serological survey against SARS-CoV-2 infections in cats **(A)** and dogs **(B)** and the amount of analyzed samples in China. Different colors represent corresponding range of sampling numbers in each province. Yellow star represents seropositive case (*n* = 1) determined by ELISA and colloidal gold testing strips.

An intensive sampling campaign was organized in seven geographic regions of China to collect blood samples from companion animals (Figure 1, Table 1). Animal samples were collected over a 3-month period (February 1 to April 18, 2021), mainly from cats and dogs but also from rabbits, hedgehogs, and guinea pigs. Animal hospitals were involved in the sampling campaign. Information on participating animals, including morphological parameters and clinical symptoms were recorded. Serum samples were stored at −20°C. Sera collected from SARS-CoV-2 vaccine immunized animals and unvaccinated animals were stored in this laboratory.

**Table 1:** Animal sera and SARS-CoV-2 seroprevalence against.

	**No. of antibody–positive cases**		**No. of collected samples**		**Seroprevalence (95% CI)**	
	**Cats**	**Dogs**	**Cats**	**Dogs**	**Cats**	**Dogs**
**Regions and provinces**						
**Overall**	**2**	**1**	**13397**	**7156**	**0.015% (0.0, 0.1)**	**0.014% (0.0, 0.1)**
**Eastern China**	**1**	**0**	**5089**	**2444**	**0.020% (0.0, 0.1)**	**0.000% (0.0, 0.2)**
Shanghai	0	0	623	227	0.000% (0.0, 0.6)	0.000% (0.0, 1.6)
Shandong	0	0	280	116	0.000% (0.0, 1.3)	0.000% (0.0, 3.1)
Jiangsu	0	0	2398	1223	0.000% (0.0, 0.2)	0.000% (0.0, 0.3)
Anhui	0	0	304	196	0.000% (0.0, 1.2)	0.000% (0.0, 1.9)
Zhejiang	1	0	1146	498	0.000% (0.0, 0.5)	0.000% (0.0, 0.7)
Fujian	0	0	125	56	0.000% (0.0, 2.9)	0.000% (0.0, 6.4)
Jiangxi	0	0	213	128	0.000% (0.0, 1.7)	0.000% (0.0,2.8)
**Central China**	**1**	**0**	**1109**	**789**	**0.090% (0.0, 0.5)**	**0.000% (0.0, 0.5)**
Hubei	1	0	267	148	0.375% (0.0, 2.1)	0.000% (0.0, 2.5)
Hunan	0	0	350	296	0.000% (0.0, 1.0)	0.000% (0.0, 1.2)
Henan	0	0	492	345	0.000% (0.0, 0.7)	0.000% (0.0, 1.1)
**Northern China**	**0**	**1**	**2987**	**1860**	**0.000% (0.0, 0.1)**	**0.054% (0.0, 0.3)**
Beijing	0	1	2199	1420	0.000% (0.0, 0.2)	0.070% (0.0, 0.4)
Tianjin	0	0	314	121	0.000% (0.0, 1.2)	0.000% (0.0, 3)
Hebei	0	0	426	265	0.000% (0.0, 0.9)	0.000% (0.0, 1.4)
Shanxi	0	0	45	53	0.000% (0.0, 7.9)	0.000% (0.0, 6.7)
Inner Mongolia	0	0	3	1	0.000% (0.0, 70.8)	0.000% (0.0, 97.5)
**Southern China**	**0**	**0**	**2672**	**1188**	**0.000% (0.0, 0.1)**	**0.000% (0.0, 0.3)**
Guangdong	0	0	2616	1144	0.000% (0.0, 0.1)	0.000% (0.0, 0.3)
Hainan	0	0	56	44	0.000% (0.0, 6.4)	0.000% (0.0, 8)
**Southwest**	**0**	**0**	**1090**	**591**	**0.000% (0.0, 0.3)**	**0.000% (0.0, 0.6)**
Sichuan	0	0	671	364	0.000% (0.0, 0.5)	0.000% (0.0, 1)
Yunnan	0	0	17	20	0.000% (0.0, 19.5)	0.000% (0.0, 16.8)
Chongqing	0	0	402	207	0.000% (0.0, 0.9)	0.000% (0.0, 1.8)
**Northwest**	**0**	**0**	**341**	**215**	**0.000% (0.0, 1.1)**	**0.000% (0.0, 1.7)**
Xinjiang	0	0	7	10	0.000% (0.0, 41)	0.000% (0.0, 30.8)
Qinghai	0	0	13	7	0.000% (0.0, 24.7)	0.000% (0.0, 41)
Shannxi	0	0	273	150	0.000% (0.0, 1.3)	0.000% (0.0, 2.4)
Gansu	0	0	48	48	0.000% (0.0, 7.4)	0.000% (0.0, 7.4)
**Northeast**	**0**	**0**	**109**	**69**	**0.000% (0.0, 3.3)**	**0.000% (0.0, 5.2)**
Jilin	0	0	109	69	0.000% (0.0, 3.3)	0.000% (0.0, 5.2)
**Gender**						
**Overall**	**2**	**1**	**13397**	**7156**	**0.015% (0.0, 0.1)**	**0.014% (0.0, 0.1)**
Female	0	0	7617	4015	0.000% (0.0, 3.3)	0.000% (0.0, 0.1)
male	2	1	5610	3101	0.036% (0.0, 0.1)	0.032% (0.0, 0.2)
unknown information	0	0	170	40	0.000% (0.0, 2.1)	0.000% (0.0, 8.8)
**Age, year**						
**Overall**	**2**	**1**	**13397**	**7156**	**0.015% (0.0, 0.1)**	**0.014% (0.0, 0.1)**
0–3	2	1	10550	3932	0.019% (0.0, 0.1)	0.025% (0.0, 0.1)
3–6	0	0	1562	1418	0.000% (0.0, 0.2)	0.000% (0.0, 0.3)
6–10	0	0	572	941	0.000% (0.0, 0.6)	0.000% (0.0, 0.4)
≥10	0	0	402	749	0.000% (0.0, 0.9)	0.000% (0.0, 0.5)
unknown information	0	0	311	116	0.000% (0.0, 1.2)	0.000% (0.0, 3.1)
**Month**						
**Overall**	**2**	**1**	**13397**	**7156**	**0.015% (0.0, 0.1)**	**0.014% (0.0, 0.1)**
Feb.	0	0	4111	2192	0.000% (0.0, 0.1)	0.000% (0.0, 0.2)
Mar.	2	0	8794	4659	0.023% (0.0, 0.1)	0.000% (0.0, 0.1)
Apr.	0	1	492	305	0.000% (0.0, 0.7)	0.328% (0.0, 1.8)

### Detection of SARS-CoV-2 antibodies by indirect enzyme-linked immunosorbent assay (ELISA)

SARS-CoV-2 antibodies were tested by indirect ELISA. Briefly, ELISA plates were coated with 200 ng/well of recombinant SARS-CoV-2 RBD protein. After saturation with 5% skimmed milk at 37 °C for 2 h, ELISA plates were washed 3 times with PBST and incubated with 100 μL of serum samples (1:100) at 37 °C for 1 h. Horseradish peroxidase-conjugated SAP (1:5000) (Bersee, Beijing, China) was used as secondary antibody. After revealing the assay with TMB substrate/H_2_SO_4_, the optical densities (ODs) were measured at 450 nm to compare the level of anti- RBD antibodies in each sample. The cut-off values were determined as the mean value of seronegative samples against SARS-CoV-2 plus 3 times of the Standard Deviations.

### Preparation of colloidal gold-based immunochromatographic strip

The colloidal was prepared using trisodium citrate method (30, 31). Briefly, 1 mL 1% chloroauric acid (Sigma–Aldrich Corporation, St. Louis, MO, USA) and 1.6 mL 1% trisodium citrate solution (Sigma–Aldrich Corporation, St. Louis, MO, USA) were added into 100 mL boiling water, kept on boiling for 5 min until the solution color changed to red, then cooled down to room temperature (RT). The quality of prepared colloidal gold was tested by UV–vis absorption spectra (Thermo Fisher Scientific, Rockford, IL, USA) and transmission electron microscope (TEM) H-600 (Hitachi High-Tech Corporation, Tokyo, Japan). S protein, purchased from Sino Biological Inc. (Beijing, China), was incubated with the colloidal gold solution at RT for 30 min, followed by centrifugation at 4°C 12,000 x *g* for 30 min; discarded the supernatant, and resuspended the pellet with boric acid buffer containing 1% BSA (Sigma–Aldrich Corporation, St. Louis, MO, USA) to obtain the colloidal gold conjugated S protein. The immunochromatographic strips were prepared as described previously (30) with the modifications as follows. Colloidal gold conjugated S protein was dispensed on fiberglass pad, staphylococcal protein A (SPA, Bersee, Beijing, China) and mouse-anti-S protein monoclonal antibody were coated on test and control area of nitrocellulose membrane, respectively.

### Detection of SARS-CoV-2 antibodies by using colloidal gold test strips

Serum samples were used at 1:100 dilutions in PBS. Sera (1:1000) collected from SARS-CoV-2 vaccine immunized mice was used as positive control. Test strips were loaded with 100 μL of sample and the results were recorded within 5 min incubation at RT. The test was considered as positive when the control line (C line) and the IgG test line (T line) appeared, and as negative when only the C line was visible. The result was invalid when no signal was observed in the C line.

### Date management and analysis

Microsoft Excel 2016 (Microsoft Corporation of Redmond, Washington, USA) was used to aggregate the information including age, medical time, species, temperature, and clinical symptoms of animals, and were used to calculate the confidence intervals (CI) of collected data. ArcGIS software (Environmental Systems Research Institute, Redlands, CA) was used to combine the collected data with geography distributions. The sensitivity, specificity, the positive predictive value and the negative predictive value of tested methods were calculated as described (32, 33). Statistical analyze was performed by SPSS software (SPSS, Inc., Chicago, IL, USA), a chi-squared test was used for comparing the difference between male and female.

## Results and discussion

One year after the first COVID-19 outbreak, a cross-sectional study was carried out to investigate the seroprevalence SARS-CoV-2 infections among domestic cats and dogs in China (Figure 1, Table 1). The minimum number of serum samples was calculated using Epitools (34) with the expected prevalence as 50 %, confidence level at 95 %, and the desired precision of 5 %. Besides, the sensitivity and specificity of ELISA method was 93.75 and 92.00%, respectively. The negative predictive value was 93.24 %, while the positive predictive value was 92.59 %. The result showed that sample size was required at least for 520. A total of 20,533 serum samples, collected from 25 provinces across 7 geographical regions, were tested for IgG antibodies against SARS-CoV-2 RBD protein using ELISA method. The colloidal gold-based immunochromatographic strip, of which the sensitivity and specificity was 89.87 and 94.67 %, respectively, while the negative and positive predictive values were 89.87 and 94.67 %, respectively, was further used to confirm the presence of SARS-CoV-2 antibodies of the positive samples screened by ELISA.

The overall seroprevalence of SARS-CoV-2 infections in the feline population was 0.015% (2/13397; 95% CI: 0.0, 0.1). Seropositive animals were only found in two of the seven regions included in the study, with a seroprevalence in Central and Eastern China estimated at 0.090% (1/1109; 95% CI: 0.0, 0.5) and 0.020% (1/5089; 95% CI: 0.0, 0.1), respectively. Narrow down to the province level, the seroprevalence was 0.375% (1/267; 95% CI: 0.0, 2.1) in Hubei (Central China) and 0.087% (1/1146; 95% CI: 0.0, 0.5) in Zhejiang (Eastern China). The seroprevalence of SARS-CoV-2 infections in the canine population was 0.014% (1/7159; 95% CI: 0.0, 0.1) in the entire nation. The seropositive sample originated from the Beijing (seroprevalence 0.070%; 1/1420; 95% CI: 0.0, 0.4) in Northern China (seroprevalence 0.054%; 1/1860; 95% CI: 0.0, 0.3) (Figure 1, Table 1).

The SARS-CoV-2 seroprevalence in the feline and canine populations in China was lower than the seroprevalence reported in several European countries, which ranged from 0.4 to 3.5% in cats and 0.2 to 3.3% in dogs (13–17). These results are expected given the long period distance from the peak outbreak of COVID-19 and the short duration of the serological response in cats. Furthermore, the human infection rate for SARS-CoV-2 was estimated at only 0.03% in China, a value 880- to 1200-fold lower than those documented in Europe. This low level of human infections in China may represent a low risk of exposure for companion animals, since SARS-CoV-2 infections in cats and dogs are considered to be favored by close contact with people with COVID-19 (35–37).

The seropositive animals were located in Wuhan, the most severely affected area of China, and Beijing, where sporadic cases were detected in the first quarter of 2021. Seropositive animals were also detected in Hangzhou, despite no reported indigenous cases of SARS-CoV-2 infections. Only one of the seropositive animals exhibited SARS-CoV-2 symptoms as diarrhea at the sampling date (Table 1, Supplementary Table S1). Frequent contact with COVID-19 patients or asymptomatic infected people is the leading cause of SARS-CoV-2 transmission from humans to animals (38–41). Until the beginning of April, 2022, the infection rate of COVID-19 was estimated at 0.65% (941,545/1,443,497,378) among humans in China. Under this condition, pet cats and dogs are in a low-risk state for a long time, these seropositive cases could be the outcome of an unusual long-standing serological response, a recent contamination, or a persistent infection. Despite a risk of “reservoir” for the virus, it was not possible to test the infectious status of these animals.

Although not statistically relevant (*p* = 0.079), it is noteworthy that only males (3/20533) were found seropositive in our serum collection that included samples of both genders (male: 42.4%, 8711/20553; female: 56.7%, 11632/20553), raising the question of a possible difference in sensitivity between sexes. Such a difference has already been documented in humans due to natural genetic differences between men and women (42, 43). Currently, the only animal species where a difference in sensitivity between males and females have been documented is the hamster (44).

Despite the limited number of seropositive samples, our data confirmed that SARS-CoV-2 can infect companion animals. In addition to the phenomenon that the coronavirus can be transmitted from humans to animals. Several studies have confirmed that human can also get infection acquired from infected animals, for example minks and pet hamster (45–47). Indicating the potential threatens of virus transmission from animals to humans. This led us to investigate the SARS-CoV-2 seroprevalence in other companion animals including rabbits and guinea pigs, two species known to be susceptible to SARS-CoV-2 (48–50). Hedgehog sera were also tested. The susceptibility of this species to SARS-CoV-2 is unknown, but hedgehog is considered a companion animal in China. As expected by the limited number of samples available, the 39 sera tested were all found negative for SARS-CoV-2 antibodies (Supplementary Table S2).

In conclusion, the transmission of SARS-CoV-2 to companion animals was limited in China, likely associated with a faster and better control of the disease epidemics. However, companion animals should be regarded as potential risk of back-transmission to humans, given the high potential for adaptation of RNA viruses. Continuous surveillance of antibody prevalence against SARS-CoV-2 in companion animals is helpful for better understanding the circulation of the virus in pet populations, and the effects of the virus on companion animals.

## Data availability statement

The original contributions presented in the study are included in the article/Supplementary material, further inquiries can be directed to the corresponding author.

## Ethics statement

The animal study was reviewed and approved by Institutional Animal Care and Use Committee of Zhengzhou University.

## Author contributions

AW and GZ contributed to conception and design of the study. XZ, YC, YS, HL, PD, and JY performed the investigation and statistical analysis. AW and XZ wrote the first draft of the manuscript. All authors contributed to manuscript revision, read, and approved the submitted version.

## Funding

This research was funded by the National Nature Science Foundation of China (NSFC) (grant number 32072944), Consulting Research Project of Chinese Academy of Engineering [grant numbers 2020-XY-76 (2020-KYGG-03-02)], 1125 Talent Gathering Plan Project of Zhengzhou, Key Projects of Zhengzhou University, and Key Science and Technology Projects of Henan Province (grant number 201100310100).

## Conflict of interest

Authors YC, YS, HL, and PD were employed by Henan Zhongze Biological Engineering Co., Ltd. The remaining authors declare that the research was conducted in the absence of any commercial or financial relationships that could be construed as a potential conflict of interest.

## Publisher's note

All claims expressed in this article are solely those of the authors and do not necessarily represent those of their affiliated organizations, or those of the publisher, the editors and the reviewers. Any product that may be evaluated in this article, or claim that may be made by its manufacturer, is not guaranteed or endorsed by the publisher.
